# Structural and Phylogenetic Studies with MjTX-I Reveal a Multi-Oligomeric Toxin – a Novel Feature in Lys49-PLA_2_s Protein Class

**DOI:** 10.1371/journal.pone.0060610

**Published:** 2013-04-03

**Authors:** Guilherme H. M. Salvador, Carlos A. H. Fernandes, Angelo J. Magro, Daniela P. Marchi-Salvador, Walter L. G. Cavalcante, Roberto M. Fernandez, Márcia Gallacci, Andreimar M. Soares, Cristiano L. P. Oliveira, Marcos R. M. Fontes

**Affiliations:** 1 Depto. de Física e Biofísica, Instituto de Biociências, Universidade Estadual Paulista–UNESP, Botucatu, SP, Brazil; 2 Depto. de Farmacologia, Universidade Estadual Paulista – UNESP, Botucatu, SP, Brazil; 3 Fundação Oswaldo Cruz – FIOCRUZ Rondônia and Centro de Estudos de Biomoléculas Aplicadas – CEBio, Universidade Federal de Rondônia – UNIR, Porto Velho, RO, Brazil; 4 Depto. de Física Experimental, Instituto de Física, Universidade de São Paulo – USP, São Paulo, SP, Brazil; Weizmann Institute of Science, Israel

## Abstract

The mortality caused by snakebites is more damaging than many tropical diseases, such as dengue haemorrhagic fever, cholera, leishmaniasis, schistosomiasis and Chagas disease. For this reason, snakebite envenoming adversely affects health services of tropical and subtropical countries and is recognized as a neglected disease by the World Health Organization. One of the main components of snake venoms is the Lys49-phospholipases A_2_, which is catalytically inactive but possesses other toxic and pharmacological activities. Preliminary studies with MjTX-I from *Bothrops moojeni* snake venom revealed intriguing new structural and functional characteristics compared to other bothropic Lys49-PLA_2_s. We present in this article a comprehensive study with MjTX-I using several techniques, including crystallography, small angle X-ray scattering, analytical size-exclusion chromatography, dynamic light scattering, myographic studies, bioinformatics and molecular phylogenetic analyses.Based in all these experiments we demonstrated that MjTX-I is probably a unique Lys49-PLA_2_, which may adopt different oligomeric forms depending on the physical-chemical environment. Furthermore, we showed that its myotoxic activity is dramatically low compared to other Lys49-PLA_2_s, probably due to the novel oligomeric conformations and important mutations in the C-terminal region of the protein. The phylogenetic analysis also showed that this toxin is clearly distinct from other bothropic Lys49-PLA_2_s, in conformity with the peculiar oligomeric characteristics of MjTX-I and possible emergence of new functionalities inresponse to environmental changes and adaptation to new preys.

## Introduction

Snakes are one of the major groups of the Squamata reptilian order, with more than 3300 extant and extinct species already identified by the scientific community [1]. Many of these animals are venomous and represent an important public health problem in rural areas of Asia, Africa and Latin America. Recently, it was attested that the mortality caused by snakebites is higher than other neglected tropical diseases, such as dengue haemorrhagic fever, cholera, leishmaniasis, schistosomiasis and Chagas disease [2]. This fact has attracted massive attention from the scientific community resulting in the publication of some important articles and reviews about the real impact of the snakebites on health services [2], [3], [4] and, recently, snakebite accidents were classified as a neglected disease by the World Health Organization (WHO) [3]. Among the venomous snakes, the world-widespread Viperidae family is one of the most harmful groups with respect to snake envenoming, especially in Asia and Latin America [3], [5]. In Latin America, the *Bothrops* viperid genus is particularly important since these animals are responsible for 85% of all ophidian accidents reported in this geographic area [6], [7]. One of the main components of bothropic and other snake venoms are the phospholipases A_2_, enzymes which are able to promote Ca^2+^-dependent hydrolysis of *sn-*2 acyl groups of membrane phospholipids, releasing free fatty acids and lysophospholipids [8]. A subgroup of these proteins, the Lys49-phospholipases A_2_ (PLA_2_s), are catalytically inactive due to the lack of Ca^2+^ coordination related to the natural mutations Tyr28→Asn and Asp49→Lys [9], [10], but, in association with metalloproteases, may cause permanent tissue loss, disability and even require limb amputation due to local myonecrosis inefficiently neutralized by serum therapy [5].

Experiments based on electrophoresis, spectroscopy [11], [12], crystallography [13], [14], [15], [16] small angle X-ray scattering [17] and dynamic light scattering [10] have brought important insights into the structural features of these molecules demonstrating that the bothropic Lys49-PLA_2_s are dimeric in solution. Crystallographic studies also revealed that these proteins have a dimeric structure and a biological unit held by contacts between the tips of β-wing segments and N-terminal α-helices from both monomers [13]. The biological significance of this dimeric conformation was straightened by Ward *et al*. (2002), whose work demonstrated the occurrence of a fluorescence signal probably originated from the interaction between the Lys49-PLA_2_-conserved residues Trp77 in a solution containing the toxin BthTX-I from *Bothrops jararacussu*. More recently, the crystal structures of three Lys49-PLA_2_s complexed to suramin and α-tocopherol were solved in an “alternative” dimeric assembly in contrast to the previous conventional form. The alternative dimer accommodates appropriately the hydrophobic segments of these ligands and presents a larger interfacial area with more negative free energy compared to the conventional dimeric form. Since it is possible to obtain the same alternative dimer in the unit cells for all solved structures to date, this choice seems to be the correct biological conformation for the Lys49-PLA_2_s [15], [18]. Small angle X-ray scattering experiments and molecular dynamic simulations with BthTX-I also show that this alternative dimer is the most probable configuration of this protein in solution [17]. Structural, functional and site-direct mutagenesis studies pointed out that the C-terminal region of Lys49-PLA_2_s (residues 115–129) is mainly responsible for their myotoxic activity [19], [20], [21], [22], [23], [24]. More recently, a specific myotoxic site for bothropic Lys49-PLA_2_s composed of two residues from C-terminal region (Lys115 and Arg118) and one from N-terminal region (Lys20) was proposed [18].

In contrast with these well-established structural and functional data for different bothropic Lys49-PLA_2_s, preliminary structural and functional studies with MjTX-I (myotoxin-I) from *Botrops moojeni* venom revealed intriguing new results. Electrophoresis experiments with a purified fraction of MjTX-I showed several oligomeric conformations [25] and its crystal structure revealed a tetrameric conformation composed by two “conventional” dimers [26]. Moreover, the MjTX-I myotoxicity measured by plasma creatine kinase activity is significantly lower than other Lys49-PLA_2_s [27]. In the light of these new results, we performed a very comprehensive study with MjTX-I using different techniques, including crystallography, analytical size-exclusion chromatography, dynamic light scattering, small angle X-ray scattering, myographic studies, bioinformatics and molecular phylogenetic analyses. The results obtained indicated that MjTX-I is probably a unique Lys49-PLA_2_, with a special capacity for adopting diverse oligomeric forms. These data reinforce the importance of quaternary assembly of Lys49-PLA_2_s to their myotoxic activity and add new elements to the functional mechanisms and evolution of these and other related molecules.

## Materials and Methods

### Ethics

Institutional Animal Care and Use Committee (Institute of Biosciences - Sao Paulo State University) approved this study under the number 033/05. Animal procedures were in accordance with the guidelines for animal care prepared by the Committee on Care and Use of

Laboratory Animal Resources, National Research Council, USA.

### MjTX-I purification

MjTX-I was isolated from *Bothrops moojeni* venom by ion-exchange chromatography in HiTrap CM Sepharose Fast Flow (5 ml; GE Healthcare™) equilibrated with 0.05 M ammonium bicarbonate buffer pH 8.0. Elution started with this buffer, followed by a gradient from 0.05 to 0.5 M ammonium bicarbonate at 20 °C as previously described [25], [28]. The purity of the MjTX-I eluted fraction was analyzed by 13% SDS-PAGE gel electrophoresis followed by Coomassie Blue staining.

### Crystallization trials

Initially, a lyophilized sample of MjTX-I was dissolved in ultra-pure water at a concentration of 12.0 mg.ml^−1^. The crystallization experiments were performed using the sparse matrix method [29] and the hanging drop vapor diffusion technique [30]. 1 µl of protein and 1 µl reservoir drop were mixed and equilibrated against 500 µl of the same precipitant solution. After approximately 350 days at 291 K, crystals appeared in a solution containing 0.15 M MgCl_2_, 32% (w/v) polyethylene glycol (PEG) 4000 and 0.1 M Tris-HCl pH 8.5 as described previously [26].

### X-ray data collection and data processing

X-ray diffraction data were collected using a wavelength of 1.421 Å at a synchrotron-radiation source (MX1 beamline – Laboratório Nacional de Luz Sincrotron, LNLS, Campinas, Brazil) with a MAR CCD™ imaging-plate detector (MAR Research™). The crystals submitted to X-ray diffraction experiments were held in appropriate nylon loops and flash-cooled in a stream of nitrogen at 100 K. The best data set (150 images; d_detector_  = 80 mm; Δφ = 1°) was processed at 2.49 Å resolution using the HKL program package [31]. X-ray diffraction data and processing statistics are presented in Table 1.

**Table 1:** X-ray data collection and refinement statistics.

Unit cell (Å,^o^)	a = 57.6, b = 125.9, c = 65.3, β = 106
Space group	C2
Resolution (Å)	33.44 – 2.49 (2.55 – 2.49)^a^
Unique reflections	15300 (1541)^a^
Completeness (%)	98.0 (98.9)^a^
*I/σ* (*I*)	20.64 (4.69)^a^
Redundancy	3.1 (3.1)^a^
Molecules in ASU	4
Matthews coefficient V_M_ (Å^3^Da^−1^)	2.12
*R_merge_* b (%)	5.7 (22.0)^a^
*R_cryst_*	25.13
*R_free_*	26.27
Number of non-hydrogen atoms	
Protein	3544
Waters	113
PEG molecule	3
Mean B-factor (Å^2^)^c^	
Overall	50.89
Ramachandran plot (%)^d^	
Residues in favored region	95.1
Residues in outlier region	1.8

a Numbers in parenthesis are for the highest resolution shell.

b *R_merge_* = Σ*_hkl_*[Σ_i_(*I_hkl,i_*−<*I_hkl_*>|)]/Σ*_hkl_*, <*I_hkl_*>, where *I_hkl,i_* is the intensity of an individual measurement of the reflection with Miller indices *h*, *k* and *l*, and <*I_hkl_*>s the mean intensity of that reflection. Calculated for *I>*−*3σ (I)*.

c Calculated with CNS program [35].

d Calculated with MolProbity program [37].

### Structure determination and refinement

The MjTX-I crystal structure was solved by the Molecular Replacement Method using the program MOLREP [32] from CCP4 package v.6.1.13 [33] and the all atom coordinates of PrTX-II monomer A (PDB access code 1QLL) [34] as the search model. After a cycle of rigid body refinement using CNS v.1.2 program [35], the resulting electron density map was used for modeling side chains corresponding to the MjTX-I amino acid sequence. The modeling process was performed by manual rebuilding using program Coot v.0.7 [36]. Polyethylene glycol (PEG) 4000 and solvent molecules were added by the programs CNS v.1.2 and Coot v.0.7, respectively. Due to the lack of electron density in some regions of the model, the following side chains of amino acid residues were not modeled: monomer A: Lys 53, Lys69, Lys93, Lys115, Lys116, and Lys122; monomer B: Lys16, Lys20, Lys36, Lys57, Lys69, Lys 70, Asp76, Lys78, Glu87, Asn88, Lys116, Val119, Lys122, and Arg131; monomer C: Lys16, Lys20, Lys36, Lys69, Lys70, Lys78, Lys93, Lys115, Lys116, and Lys129; monomer D: Lys7, Gln11, Lys16, Lys20, Lys57, Leu58, Lys69, Lys70, Tyr73, Asp76, Trp77, Glu86, Asn88, Lys115, and Phe125. For the same reason, the amino acid residues Val119, Tyr120, Leu121, and Lys122 from monomer C and Asp118, Val119, Tyr120, Leu121, and Lys122 from monomer D were also completely removed from the model. MolProbity program (http://molprobity.biochem.duke.edu/) [37] was used to check the general quality of the final model. The van der Waals intermolecular interactions were detected with the software PSAIA (Protein Structure and Interaction Analyzer) [38] using a threshold of 1.5 Å. The refinement statistics and other information are shown in Table 1. All structural figures were generated using PyMOL v.1.3 program [39].

### Dynamic light scattering

The dynamic light scattering (DLS) experiments were executed with MjTX-I at 1.5 mg.mL^−1^ concentration and 18°C using a device DynaPro TITAN™ (Wyatt Technology™). Measurements were carried out with the protein dissolved in ultra-pure water or in different Tris-HCl pH 8.0 concentrations (0.5, 2.0, 5.0, 10.0, and 20.0 mM). One hundred measurements were acquired in each experiment; in the case of solutions containing buffer, the data were obtained immediately after the Tris-HCl pH 8.0 addition and two hours later. The analysis of the final data was performed with the program Dynamics v.6.10 (Wyatt Technology™).

### Analytical size exclusion chromatography

Analytical size-exclusion chromatography (SEC) was performed with the purified MjTX-I using a Superdex 75 10/300 GL (GE Healthcare™) column equilibrated with 20 mM Tris-HCl pH 8.0 at 20°C. The MjTX-I sample (V = 0.1 ml; [2 mg.ml^−1^]) was injected at a flow rate of 0.5 ml/min. The molecular weight standards were obtained from a low molecular weight gel filtration calibration kit (Sigma-Aldrich™) containing the following components: blue dextran (2000 kDa), bovine serum albumin (66 kDa), carbonic anhydrase (29 kDa), cytochrome C (12.4 kDa), and aprotinin (6.5 kDa). The retention volumes for each standard and sample were measured and used to calculate the partition coefficients (Kav), which are defined as Kav = (Vr−Vo)/(Vc−Vo), where Vr =  retention volume, Vo =  void volume (calculated based on the retention time of the blue dextran standard), and Vc =  geometric bead volume of the column. The coefficient Kav for each standard was plotted against the log of the molecular weight in order to generate a standard curve, which was used to determine the approximate molecular weight of each oligomeric species found in the experimental sample.

### Small angle X-ray scattering analysis

Small-angle X-ray scattering (SAXS) experiments were performed with MjTX-I dissolved in ultra-pure water on 20 mM Tris-HCl pH 8.0at 5.0 mg.ml^−1^ concentration. The measurements were taken at room temperature using NANOSTAR™ equipment from Bruker™, placed at the Laboratory of Crystallography at the Institute of Physics of the University of São Paulo. Scattering experiments on the liquid samples were performed using reusable quartz capillaries glued on stainless steel cases. Background intensities were obtained based on scattering by the corresponding buffers measured using the same capillaries. The data obtained by 3600 s exposure were analyzed with the package SUPERSAXS (Oliveira & Pedersen, unpublished). Experimental data are shown as intensity I(q) versus the momentum transfer *q = (4π/λ)sinθ*, where *λ* is the radiation wavelength and *2θ* is the scattering angle. After treatment, the data were normalized to absolute scale using water as the primary standard.The Indirect Fourier Transformation (IFT) was performed using the Glatter method [40] with a slightly different implementation [41]. In order to obtain better structural insights, the SAXS data were compared with the MjTX-I crystallographic structure described in this work, using the program CRYSOL [42]. As described later, since this structure was solved with four monomers in the asymmetric unit, the tetrameric, dimeric and monomeric structures could be tested and compared to the experimental data. Finally, the *ab initio* modeling was performed using the program GASBOR [43]. In this method a sequence of interconnected chains is used to represent the protein backbone. Each sphere corresponds to one amino acid and therefore the total number of spheres is identical to the number of protein residues. Starting from a spherical arrangement of the backbone the program performs a simulated annealing optimization in which the backbone three-dimensional arrangement is changed to improved fitting with the scattering data. As result, a model representing the low resolution structures of the protein is obtained.

### Myographic Studies

Adult male mice weighing 25 to 30 g were maintained under a 12 h light-dark cycle (lights on at 07:00) in a temperature controlled environment (22±2°C) for at least ten days prior to the experiments. Food and water were freely available.

Mice were killed by exsanguination after ether anesthesia. The phrenic nerve-diaphragm preparation was removed and mounted vertically under a tension of 5 g in a conventional isolated organ bath chamber containing 10 ml of physiological solution (Ringer), with the following composition (mmol/l): NaCl, 135; KCl, 5; MgCl_2_, 1; CaCl_2_, 2; NaHCO_3_, 15; Na_2_HPO_4_, 1; glucose, 11. This solution was gassed with carbogen (95% O_2_ and 5% CO_2_) and kept at 35 ± 2 °C. The preparation was attached to an isometric force transducer (Grass, FT03) coupled to a signal amplifier (Gould Systems, 13-6615-50). The experiments were recorded using a computer-based data acquisition system (Gould Systems, Summit ACQuire and Summit DataViewer). Indirect contractions were evoked by supramaximal strength pulses (0.2 Hz; 0.5 ms; 3 V), delivered by an electronic stimulator (Grass S88K) and applied on the phrenic nerve by suction electrode. Direct contractions were evoked by supramaximal pulses (0.2 Hz; 5 ms; 13 V) through a bipolar electrode positioned on opposite sides of the muscle. Experiments of direct contractions were performed in the presence of pancuronium bromide (2×10^−6^ M). The preparations were stabilized for at least 45 minutes before the toxin addition. The amplitudes of indirect and direct twitches were evaluated during 90 and 120 minutes respectively. The mean time required to reduce the twitches amplitude to 50% (t_1/2_) was determined.

Results of myographic studies are expressed as mean ± S.E. and were analyzed by Student's *t*-test. Values of *P*<0.05 were considered significant.

### Sequence alignment and phylogenetic analysis

Alignment of the selected sequences was performed by the program AMAP v. 2.0 [44]. The final alignment was used to construct a phylogenetic tree by Bayesian inference utilizing MrBayes v. 3.1.1 software [45]. Two concurrentMCMC runs of 500,000 generations were performed with four progressively heated chains, a temperature value of 0.2, tree sampling every 100 generations and a burn-in of 2500 trees. The phylogenetic tree and the lengths of its branches were visualized using the program Mesquite v.2.72 [46].

## Results

### Overall crystallographic structure of MjTX-I

**Figure 1:**
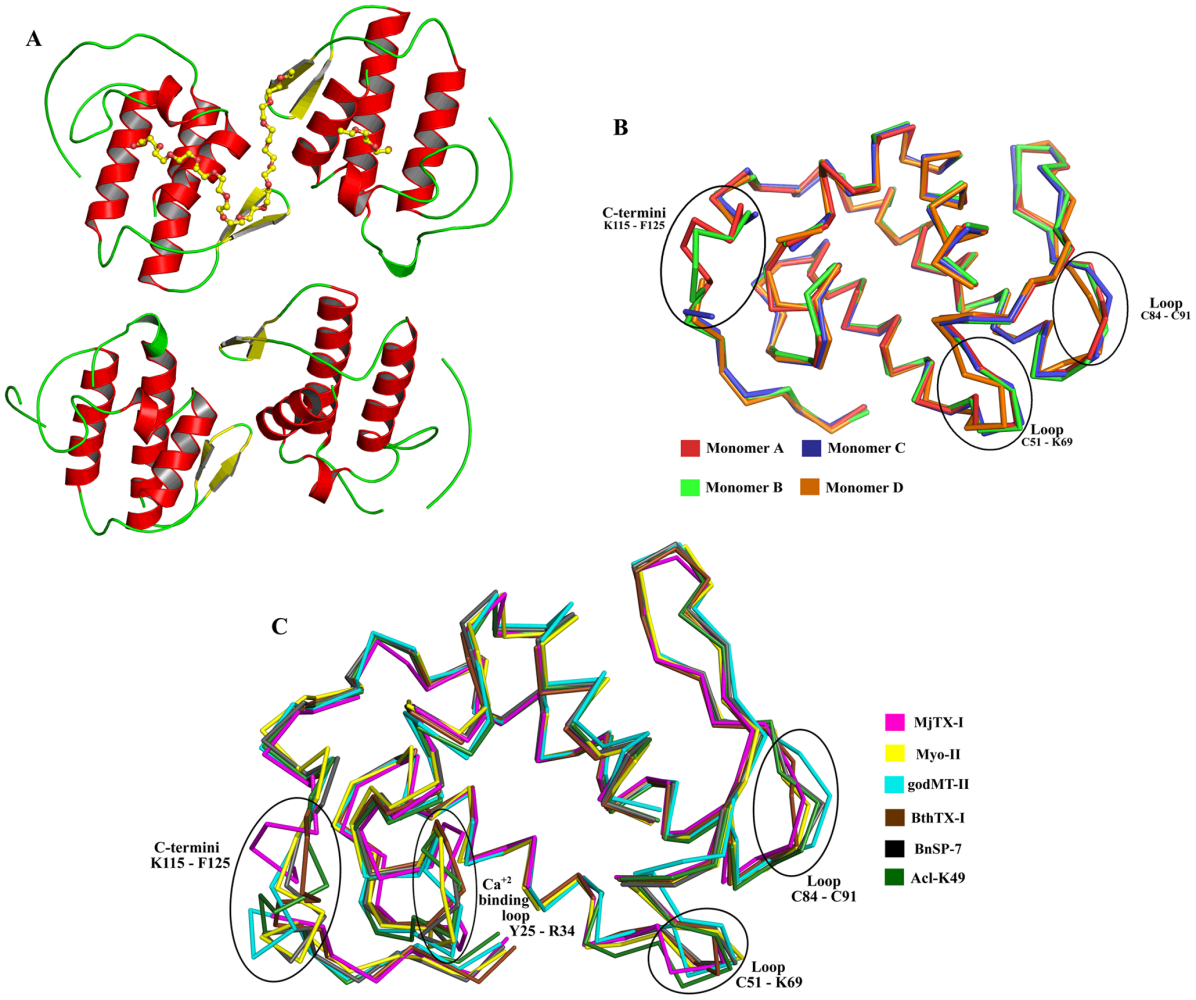
Overall crystallographic structure of MjTX-I. (A) Cartoon representation of MjTX-I structure. PEG4000 molecules are showed in sticks. (B) C^α^ superposition of monomers A (red), B (light green), C (blue) and D (orange) of MjTX-I structure highlighting the most important structural deviations between them. (C) C^α^ superposition of monomers A of MjTX-I (magenta), BnSP-7 (PDB ID 1PA0) (black) and Myo-II (PDB ID 1CLP) (yellow), monomer B of BthTX-I (PDB ID 3HZD) (brown) and overall structure of godMT-II (PDB ID 1GOD) (cyan) and Acl-K49 (PDB ID 1S8H) (dark green).

The crystal structure of MjTX-I at 2.49 Å resolution revealed an asymmetric unit (AU) containing four monomers (identified as A, B, C, and D) and C2 space group with cell constants *a = *57.6 Å, *b* = 125.8 Å, *c* = 65.3. Å, and β = 106°. As shown in the Table 1, the refinement converged to a final R_cryst_ value of 25.13% (R_free_ = 26.27%) for all data between 33.44 Å and 2.49 Å. In the final model are found 3544 non-hydrogen protein atoms, 113 water molecules and three polyethylene glycol (PEG) 4000 molecules. As in other Lys49-PLA_2_s structures, two of them are close to His48 in monomers A and B, and the third PEG 4000 molecule is sited between the interface of the monomers A and B. MjTX-I is stabilized by seven disulfide bridges and preserves the classical secondary structure elements found in this group of proteins, i.e., a N-terminal α-helix, a “short” helix, a non-functional Ca^2+^-binding loop, two anti-parallel α-helices (2 and 3), two short strands of anti-parallel β-sheet (β-wing), and a C-terminal loop (Figure 1A). The general stereochemical quality of the final MjTX-I structure is also satisfactory, since the Ramachandran plots shows that 95.1% and 98.2% of the total number of amino acid residues are presentin the favored and allowed φ/ψ angle combinations, respectively. The residues Pro90 (monomer A), Glu87, Pro123, Phe125 (monomer B), Leu55, Asp67, Lys116, Phe125 (monomer D) are found in the outlier regions of the Ramachandran plot, which corresponds to 1.8% of the total amino acid residues of the final model. These residues are sited inregions with high flexibility: C-termini (Lys116, Pro123 and Phe125) and loop segments (Leu55, Asp67, Glu87 and Pro90). The amino acid composition of the interfaces between the monomers A, B, C and D are shown as Supporting Information (Table S1).

**Figure 2:**
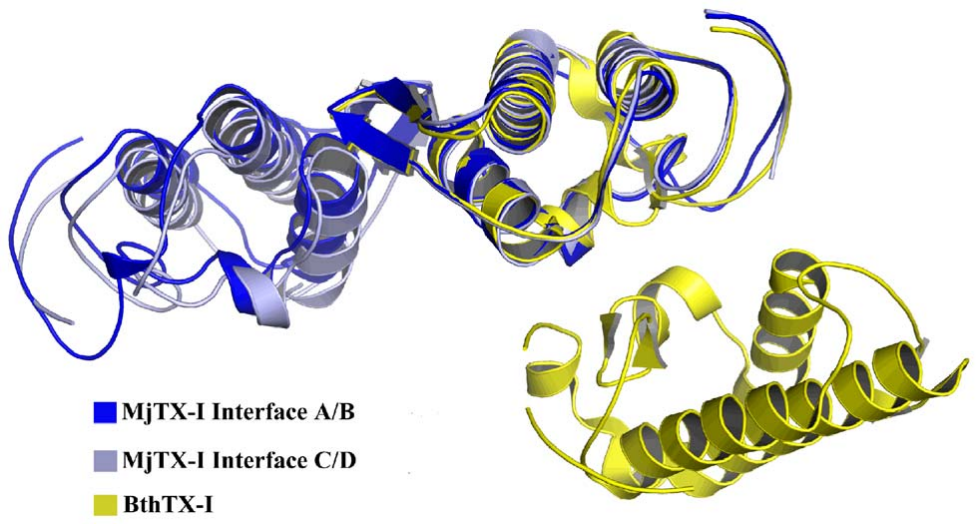
Two possible dimeric conformations for Lys49-PLA_2_s structures. The alternative dimer formed by dimer of BthTX-I chemically modified by *p*-bromophenacyl bromide (yellow) (PDB ID 3HZW) and the conventional dimerformed by interfaces of the monomers A/B (blue) and C/D (gray) in MjTX-I crystal structure.

A detailed analysis of the AU shows that the monomers are roughly disposed in a plane along the four β-wings, with the C-termini occupying external positions in relation to the protein core (Figure 1A). Interestingly, C^α^ pairwise structural superposition between these protomers indicates that the chain D is slightly distinct, with a root mean square deviation (r.m.s.d.) around 0.8 Å in relation to the other protomers (Table 2). The main structural alterations contributing to this C^α^ atom deviation are concentrated in the β-wing region of the monomer D, which is part of the interface between the chains C and D (Figure 1B). This feature of the monomer D is probably related to the different contacts observed for the A/B and C/D monomeric pairs, according to the PDBe PISA server (pdbe.org/pisa). In fact, the A/B interface presents seven hydrogen bonds and four salt bridges, whereas the C/D interface is formed by four hydrogen bonds and three salt bridges, as indicated in the (Table S2). In spite of the distinct number of contacts at the interfaces of pairs of monomers, they are formed between the same amino acid residues (Table S2). In addition, there is a PEG 4000 molecule at the A/B interface region which forms hydrophobic interactions with the ligand and the monomers, increasing the number of contacts and compacting the A/B dimer. Moreover, the absence of the PEG 4000 molecule at the interface of the C/D apparently allows a little dislocation of the monomer D and a subsequent displacement of its β-wing region in comparison to the other monomers. Furthermore, the monomers form two “conventional” dimers, similar to the conventional dimeric structure suggested for Lys49-PLA_2_s [13]. These dimers are linked by the interfaces A/B and C/D (Figure 2) presenting a large number of van der Waals contacts that contribute significantly for oligomeric stabilization (Table S1).

**Table 2:** Superposition between protomers of MjTX-I, BthTX-I (PDB ID 3HZD), BnSP-7 (PDB ID 1PA0), godMT-II (PDB ID 1GOD), Acl-K49 (PDB ID 1S8H) and Myo-II (PDB ID 1CLP) (r.m.s. deviation (Å) of C^α^ atoms).

		MjTX-I A	MjTX-I B	MjTX-I C	MjTX-I D	BthTX-I A	BthTX-I B	BnSP-7 A	BnSP-7 B	godMT-II A	Acl-K49 A	Myo-II A	Myo-II B
MjTX-I	A	–	0.8	0.6	0.8	1.0	1.2	1.2	1.2	1.4	1.4	1.5	1.5
	B	–	–	0.5	0.8	0.7	1.1	1.2	0.9	1.5	1.3	1.5	1.5
	C	–	–	–	0.8	0.8	0.9	1.0	0.9	1.4	1.2	1.4	1.4
	D	–	–	–	–	1.0	1.1	1.2	1.0	1.5	1.3	1.2	1.5

### Structural comparison of MjTX-I with other Lys49-PLA_2_s

Superposition between C^α^ atoms of the MjTX-I and protomers of several bothropic Lys49-PLA_2_s deposited in PDB resulted in an r.m.s.d. of approximately 1 Å. The same superposition including non-bothropic venoms resulted in an r.m.s.d. of approximately 1.4 Å (Table 2). The superposition between protomers of MjTX-I, BnSP-7 from *Bothrops pauloensis* (PDB ID 1PA0), Myo-II from *Bothrops asper* (PDB ID 1CLP), BthTX-I from *Bothrops jararacussu* (PDB ID 3HZD), godMT-II from *Cerrophidion godmani* (PDB ID 1GOD) and Acl-K49 from *Agkistrodon contortrix laticinctus* (PDB ID 1S8H) shows that the deviations are concentrated in the Ca^2+^-binding loop (Tyr25-Arg34), loops Cys51-Lys69 and Cys84-Cys91 and in the C-terminal region (Lys115-Phe125) (Figure 1C). C^α^ atoms superposition of this region (C-termini) between BthTX-I protomers and other Lys49-PLA_2_s resulted in an average r.m.s.d. of 1.7Å whereas the same superposition for MjTX-I protomers resulted in an average r.m.s.d. of 2.3Å (Table 3). These data show that the C-terminal regions of MjTX-I present a higher structural deviation compared to other Lys49-PLA_2_ C-termini.

**Table 3:** Superposition between C-terminal segments (K115-F125) of MjTX-I, BthTX-I (PDB ID 3HZD), BnSP-7 (PDB ID 1PA0), godMT-II (PDB ID 1GOD), Acl-K49 (PDB ID 1S8H) and Myo-II (PDB ID 1CLP) (r.m.s. deviation (Å) of C^α^ atoms).

		BnSP-7 A	BnSP-7 B	godMT-II A	Acl-K49 A	Myo-II A	Myo-II B
BthTX-I	A	2.8	0.4	2.6	2.4	2.6	2.8
	B	0.2	2.7	0.9	1.7	0.8	0.8
MjTX-I	A	2.9	2.2	2.9	2.7	3.0	3.0
	B	2.7	1.3	2.6	1.9	2.5	2.7
	C	2.3	1.2	2.2	1.1	2.6	2.0
	D	2.7	1.5	2.7	0.7	3.0	3.1

### Dynamic light scattering and analytical size exclusion chromatography

Dynamic light scattering (DLS) experiments show MjTX-I to be predominantly monomeric when dissolved in ultra-pure waterat 1.5 mg.ml^−1^ concentration, since there is a unimodal molecular distribution (Pd = 16.5%) with an average molecular weight (MW) of around 19 KDa, as calculated from a hydrodynamic radius (R_H_) value of 2.1 nm (Table 4). This result is based on the fact that the average MW of MjTX-I is approximately 14 KDa. On the other hand, the DLS measurements also indicated molecular aggregation after a gradual increase in the concentration of the Tris-HCl (the same buffer used for crystallization) and two hours of incubation (Table 4). After the incubation, MjTX-I appears to assume a dimeric form at 0.5, 2.0, and 10.0 mM Tris-HCl concentration (R_H_ = 2.7–2.8 nm; average MW ≈ 33–37 KDa), whereas at 20 mM Tris-HCl the main form of the protein is probably tetrameric (R_H_ = 3.7 nm; average MW ≈ 70 nm). Additionally, it is interesting to highlight the Tris-HCl incubated solutions present higher polidispersity percentage compared to the other samples (water or not incubated Tris-HCl conditions), indicating the formation of multi-oligomeric assemblies of the toxin.

**Table 4:** Hydrodynamic radius, polidispersity percentage and mass obtained by dynamic light scattering (DLS) experiments.

	R (nm)	MW (kDa)	% Pd	% Mass
Water	2,1	19	16,5	99,6
0,5 mM	2,1	19	19,3	99,4
0,5 mM*	2,7	33	37,6	99,8
2,0 mM	1,9	15	17,6	99,8
2,0 mM*	2,8	37	32,3	99,3
10 mM	1,9	15	15,8	99,8
10 mM*	2,8	37	28,6	99,8
20 mM	3,7	71	25,3	97,6

* After two hours of TrisHCl addition

The percent mass represents the amount of mass of the molecule with the hydrodynamic radius obtained. Measurements were carried out with the protein dissolved in ultra-pure water or in different Tris-HCl pH 8.0 concentrations (0.5, 2.0, 5.0, 10.0, and 20.0 mM). In the case of the solutions containing buffer, the data were obtained immediately after the Tris-HCl pH 8.0 addition and two hours later.

**Figure 3:**
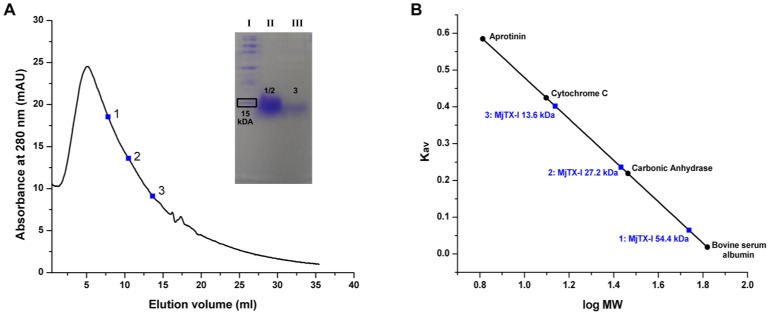
Analytical size-exclusion chromatography experiments for MjTX-I. (A) The inset shows the 13% SDS-PAGE of MjTX-I collected in different positions of the obtained peak. Lane I: unstained SDS-PAGE Standard from Bio-Rad Laboratories, Inc.; Lane II: MjTX-I collected at the elution volumes correspondent to points 1 and 2 in the chromatogram; Lane III: MjTX-I collected at the elution volume correspondent to point 3 in the chromatogram. (B) Calibration curve obtained using standard proteins shows the different oligomeric assemblies of MjTX-I in 20 mM Tris-HCl pH 8.0. The protein standards were obtained from a low molecular weight gel filtration calibration kit (Sigma-Aldrich) containing: blue dextran (2000 kDa), bovine serum albumin (66 kDa), carbonic anhydrase (29 kDa), cytochrome C (12.4 kDa) and aprotinin (6.5 kDa).

Analytical size exclusion chromatography experiment showed a broad curve of absorbance during the elution of the MjTX-I sample (Figure 3A), confirming the formation of several oligomeric species at 20 mM Tris-HCl concentration and pH 8.0. Based on a calibration curve determined using standard proteins, it is also possible to attest the occurrence of monomers, dimers and tetramers in the eluted solution (Figure 3B).

### Small angle X-ray scattering

**Figure 4:**
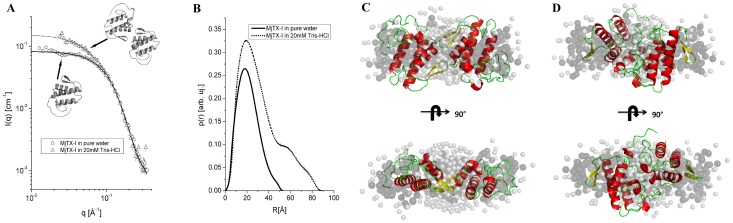
Small angle X-ray scattering experiments for MjTX-I. (A) Theoretical curve for monomeric state (continuous) corresponding to experimental SAXS data (circles), and theoretical curve for dimeric state (dashed) corresponding to experimental SAXS data (triangles). The theoretical curves were calculated by the program CRYSOL using the atomic resolution coordinates from the structures of the monomer and dimer respectively. (B) Pair distance distribution curve p(r) calculated using the IFT procedure. (C) Conventional dimer (in cartoon) superposed on the SAXS *ab initio* dummy chain model (gray transparent surface) and after 90° rotation (D). Alterative dimer (in cartoon) superposed on the SAXS *ab initio* dummy chain model and shown after 90° rotation.

Radius of gyration (R_g_) and molecular mass calculated for the main species in each SAXS experiment were1.67±0.50 nm and 18±3 KDa in ultra-pure water and 2.62±0.30 nm and 43±6 KDa in the presence of 20 mM Tris-HCl pH 8.0, respectively, underlining the significant influence of the physical-chemical environment on the oligomerization of this protein (Figure 4). It is important to observe that high-order aggregates produce noticeable scattering intensity only to very small angle around the direct X-ray beam, consequently they cannot be detected [47].

The presence of a shoulder in the p(r) curve obtained with MjTX-I in the buffered solution indicates a correlation distance within the structure which is a signature of dimeric structures. Monomeric and dimeric structures provided a very good fit for the sample in water and in buffered solution, respectively (Figure 4), supporting the monomer-dimer conversion caused by the buffer addition. In Figure 4, panels C and D present the superposition of the *ab initio* model obtained from the SAXS data and the conventional and alternative dimers [16]. The best agreement is between the *ab initio* model and the conventional dimer, whereas the superposition for the alternative dimer is unsatisfactory. Also, the alternative dimeric form provides a poor fitting of the scattering data (data not shown). Therefore, these results support the conclusion that MjTX-I does not form dimers with the alternative conformation in our experiments.

### Myographic studies

**Figure 5:**
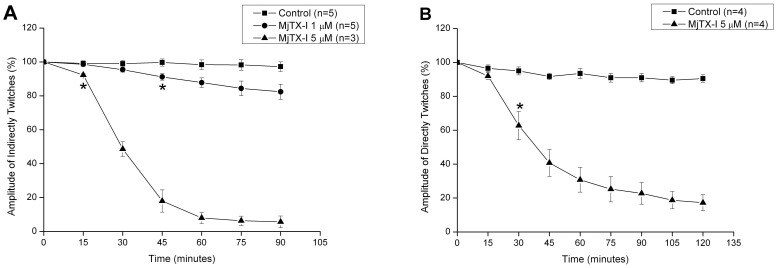
Effects of MjTX-I on indirectly (A) and directly (B) evoked twitches on isolated mouse preparations. Neuromuscular blockade was produced by 1 µM (A) and 5 µM (A, B) of MjTX-I in comparison to control. The ordinate represents the % amplitude of twitches relative to the initial amplitude. The abscissa indicates the time (min) after the addition of MjTX-I to the organ bath. Vertical bars represent mean ± SEM. * indicates the point from which there are significant differences relative to control (p<0.05).

MjTX-I induced a time- and concentration-dependent inhibition of the indirectly evoked twitches in mice phrenic diaphragm preparation (Figure 5A). At 1 µM, the toxin slightly reduced the amplitude of twitches in about 15% after 90 minutes, while at 5 µM the twitches were nearly abolished. At this same concentration (5 µM), MjTX-I also depressed directly evoked twitches in about 80% (Figure 5B). The t_1/2_ of indirectly and directly evoked twitches were not significantly different (29.6 ± 1.7 minutes; n = 3 vs. 39.5 ± 5.3 minutes; n = 4).

## Discussion

### MjTX-I may adopt different oligomeric conformations in solution

Oligomerization is a common physical property of proteins and represents a recurring theme in biological systems [48], [49]. To date, bothropic Lys49-PLA_2_s have been reported in monomeric or dimeric forms in the PDB and structural studies have demonstrated the importance of the dimeric form to expression of their myotoxic activity [16]. In addition, results obtained from bioinformatics tools demonstrated that all bothropic Lys49-PLA_2_s reported as monomeric in the crystal structures are probably dimeric in solution [10], [17]. In the light of the novel crystallographic assembly obtained for MjTX-I, several other experimental and theoretical techniques were employed to evaluate its biological significance. Initially, the PDBe PISA program [50] was not able to identify any quaternary association that might be stable in solution. According to this theoretical analysis, no quaternary assembly found in the MjTX-I crystal structure reflects the functional unit of the toxin and, consequently, only the monomeric form must be considered as the feasible biological entity. Despite the absence of oligomerization according to the PDBe PISA Complexation Significance Score (CSS), this program was able to identify important interactions between the A/B and C/D interfaces (Table S2). Then, it seems reasonable to assume that the molecular arrangement which defines the AU is not a simple crystallization artifact. This supposition is well supported by the great number of dimeric Lys49-PLA_2_s deposited in the PDB in comparison to the number of monomeric ones [10], [16], [23].

Dynamic light scattering (DLS) experiments show that MjTX-I is predominantly monomeric when dissolved in ultra-pure water, but after the addition and gradual increase of the Tris-HCl concentration, a molecular aggregation process occurs, indicating that MjTX-I may assume a dimeric or tetrameric conformation and even high-order aggregates (as demonstrated by the high polidispersity percentage and mass percentage <99% after two hours of incubation of 10 mM Tris-HCl). Thus, considering the Tris-HCl concentration at crystallization condition (100 mM) and the similar pH values in these experiments (8.0 and 8.5, respectively, in crystallization and DLS conditions), the structural arrangement of the four monomers found in the AU could reflect a possible MjTX-I physiologically relevant assembly. In agreement with the DLS results, the SAXS data also showed that MjTX-I oligomerizes in buffered solution. Further, the analytical size exclusion chromatography experiment (Figure 3) confirmed that MjTX-I has a remarkable tendency to oligomerization, confirming the formation of several oligomeric species at 20 mM Tris-HCl concentration and pH 8.0. Indeed, it was reported based on gel filtration experiments that this toxin presents a different proportion of oligomeric forms [25].

It has been observed that in several oligomeric proteins the frequency of charged and polar residues at the oligomeric interfaces is higher compared to their core regions, while hydrophobic residues are less frequent at the interfaces [51], [52], [53]. As shown in Table S1, eight polar or charged residues (∼29%) of interdimeric interfaces (A/C, B/D and A/D) are exclusively found in MjTX-I sequence. Remarkably, at least one of these residues are involved in 50% of van der Waals contacts between the interdimeric interfaces, highlighting the important role of the exclusive MjTX-I residues in the formation of the tetrameric oligomeric assembly not observed in any other Lys49-PLA_2_s.

In conclusion, we can state that this protein may adopt different oligomeric under conditions close physiological.

### MjTX-I biological oligomeric conformation and structural evidences for its lower myotoxic activity

**Figure 6:**
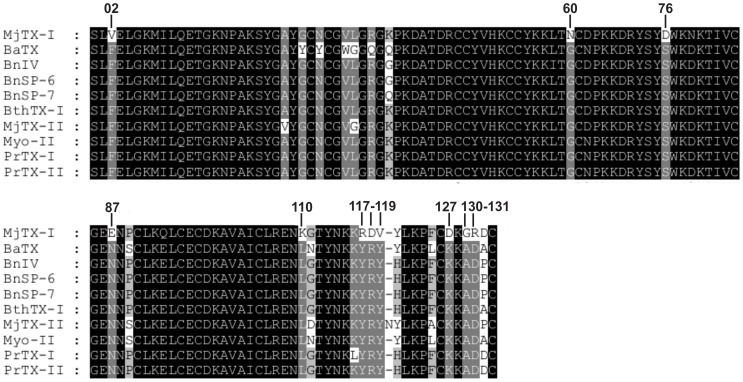
Amino acid alignment of Lys49-PLA_2_s from venom of *Bothrops* genus. The numbers upside of the alignment correspond to residues that are exclusively from MjTX-I. **BaTX**: Lys49-PLA_2_ from *Bothrops alternatus* venom (NCBI GI: 292630846); **BnIV**: Lys49-PLA_2_ from *Bothrops pauloensis* venom (NCBI GI: 333361256); **BnSP-6**: Lys49-PLA_2_ from *Bothrops pauloensis* venom (NCBI GI:49258448); **BnSP-7**: Lys49-PLA_2_ from *Bothrops pauloensis* venom (NCBI GI: 239938675); **BthTX-I**: Bothropstoxin-I from *Bothrops jararacussu* venom (NCBI GI: 51890398); **MjTX-I**: Myotoxin-I from *Bothrops moojeni* venom (NCBI GI: 17368325); **MjTX-II**: Myotoxin-II from *Bothrops moojeni* venom (NCBI GI: 62738542); **Myo-II**: Myotoxin-II from *Bothrops asper* venom (BaspTX-II) (NCBI GI: 166215047); **PrTX-I**: Piratoxin-I from *Bothrops pirajai* venom (NCBI GI: 17433154); **PrTX-II**: Piratoxin-II from *Bothrops pirajai* venom (NCBI GI: 17368328). The *Bothrops pauloensis* species was recently reclassified as *Bothropoides pauloensis* [64].

In contrast with recently propositions supporting the alternative dimer [15], [17], [18], our crystallographic and SAXS analyses indicated that the probable dimeric conformation of MjTX-I in solution is similar to the conventional oligomeric form [13], [21]. The crystal structure is formed by two conventional dimers and the comparison between the experimental data and the theoretical scattering curves of SAXS experiments indicate that the conventional dimer is the predominant configuration found in solution (Figure 4). Additionally, an analysis of the sequence alignment of MjTX-I with other bothropic Lys49-PLA_2_s (Figure 6) also supports the results obtained by SAXS and crystallography techniques. MjTX-I has a Tyr→Val mutation at position 119 compared to other bothropic Lys49-PLA_2_s, which is essential for the stabilization of the alternative dimer configuration and for their myotoxic activity. Tyr119 residues from both chains form a hydrogen bond when a ligand is present at their hydrophobic channels or His48 region, inducing the correct structural arrangement of the myotoxic site via C-termini organization [18], [54]. In the alternative conformation both C-terminal regions lie in close proximity and form the proposed myotoxic site [18], while in the conventional dimer the C-termini are in opposite sides of the dimers [13]. Furthermore, MjTX-I crystallographic structure presents a higher deviation of C-terminal region in comparison with other Lys49-PLA_2_s (Table 3) and the sequence of this regionshows only partial conservation, with the occurrence of other mutations in addition to the Tyr119→Val cited above (e.g. Tyr117→Arg, Lys127→Asp and Asp130→Arg - Figure 6). These mutations may also affect the muscle damage activity due to the change of conserved residues to other with very distinct physical-chemical properties. Indeed, site-directed mutagenesis studies showed that these C-terminal residues play an important role in the myotoxic activity of the Lys49-PLA_2_s [20], [24].

All these structural data are in agreement with functional data obtained by us and also described in previous studies [27]. In our functional studies, isolated neuromuscular preparations have been used for discrimination between neurotoxic and myotoxic effects of snake venoms or isolated toxins [55], [56]. While neurotoxicity causes only the loss of the indirect twitches, myotoxicity induces depression of both direct and indirect twitches [54]. Thus, the observation that MjTX-I simultaneously depressed both indirectly and directly evoked twitches in phrenic–diaphragm preparation may be taken as an indicative of the myotoxic effect of this protein. Similar results, i.e. the blockage of indirect and direct twitches, were previously described for BthTX-I [57]. However, the myotoxic effect of MjTX-I is significantly weaker in comparison to other bothropic Lys49-PLA_2_s [58]. While the blockage of the indirect twitches induced by MjTX-I (1 µM) did not reach 50% in 90 minutes, the reported t_1/2_ for BthTX-I, PrTX-I from *Bothrops pirajai* and MjTX-II from *Bothrops moojeni* under the same experimental conditions were 40.3 ± 3.5 min, *n* = 8; 49.0 ± 6.9 min, *n* = 6 and 35.2±2.0 min, *n* = 8, respectively [58].

Based on our functional and structural data we suggest that the lower myotoxity activity of MjTX-I in comparison to other Lys49-PLA_2_s is associated with its oligomeric conformation (conventional dimer) and also due to its capacity to adopt different oligomeric conformations depending on the physical-chemical environment. These data corroborate previous results which demonstrated that decrease in myotoxicity is associated with the formation of high molecular weight complexes [25].

### MjTX-I evolutionary aspects

**Figure 7:**
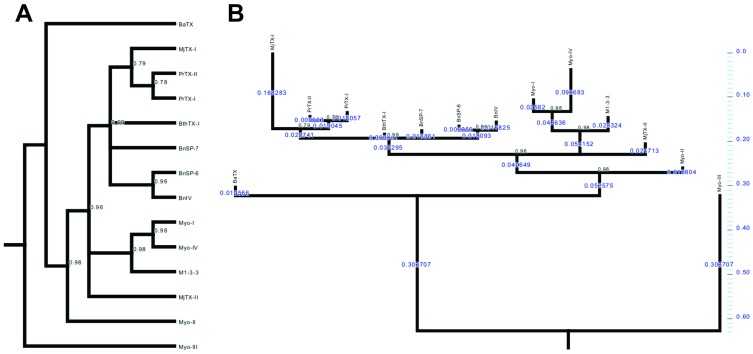
Phylogenetic tree of bothropic Lys49-PLA_2_s visualized in square form (A) and computed branch lengths (in blue) (B). Posterior probability values after 1,000,000 cycles are indicated in internodes. Internodes with a posterior probability value less than 0.75 were collapsed. Minimum e-value is 3.10^−47^. The sequences used to perform this phylogentic tree are the same sequences used in amino acid alignment of Lys49-PLA_2_s (see the legend for Figure 6) and **M1-3-3**: Lys49-PLA_2_ from *Bothrops asper* venom (NCBI GI: 6492260); **Myo-I**: Myotoxin-I from *Bothrops atrox* venom (NCBI GI: 82201805); **Myo-IV**: Myotoxin IV from *Bothrops asper* venom (NCBI GI: 166216293). Outgroup: **Myo-III**: Asp49-PLA_2_ Myotoxin III from *Bothrops asper* venom (NCBI GI: 166214965).

The results presented above strongly suggest that MjTX-I is a unique Lys49-PLA_2_, which is able to adopt a different dimeric conformation compared to other Lys49-PLA_2_s and even a tetrameric assembly formed by the association of two dimers. In the light of this variability in oligomeric structure inherent to MjTX-I, it is possible to raise interesting questions on the evolution and biological functions of this toxin. As previously mentioned, MjTX-I has lower myotoxic activity compared to other Lys49-PLA_2_s probably due to: i) high structural deviation and mutation of some residues in the C-terminal region; ii) formation of multi-oligomeric assemblies; and iii) formation of conventional dimer. In order to examine these hypotheses from an evolutionary perspective, we performed a baeysian phylogenetic analysis using bothropic Lys49-PLA_2_s amino acid sequences deposited in the NCBI protein database (Figure 7). As observed previously, the bothropic Lys49-PLA_2_s nest in a clade [59]. Despite its peculiar characteristics, MjTX-I is the sister group of PrTX-I and PrTX-II, proteins that present an alternative dimeric conformation (Figure 7). However, analyzing this phylogenetic tree considering the evolutionary distances of the Lys49-PLA_2_s to their common ancestral, MjTX-I presents the largest branch length of the tree, accumulating more sequence differences from the common ancestor. This evolutionary feature could reflect the peculiar oligomeric characteristics of MjTX-I and may be a product of the accelerated evolution of these enzymes [60].

The oligomerization of multiple, identical subunits is a simple way of forming large, functional structures in a genetically economical manner [61]. In terms of venom evolution, the core set of venom genes found in the common ancestor of toxicoferans have evolved to form the more complex reptile venoms, improved posteriorly by toxin recruitment and neofunctionalisation events, including the assembling of covalently or non-covalently-linked multi-unit toxins [62], [63]. In this regard, it is reasonable to suppose that oligomeric forms of MjTX-I can be associated to the emergence of new functionalities. Hence, the oligomeric conformation of MjTX-I may reflect an intermediate molecular state of this protein in a continuous evolutionary process that may be a response to environmental variation and adaptation to new preys.

## Conclusion

MjTX-I may adopt different oligomeric conformations depending on the physical-chemical environment as demonstrated by different techniques. Basically, this protein adopts the form of one or two dimers in the conventional configuration. Consequently, it is likely that the oligomerization states presented by MjTX-I in comparison to other Lys49-PLA_2_s could be intrinsically related to its biological functions. In evolutionary terms, the oligomeric forms of MjTX-I may be also associated to the emergence of new functionalities, since these assemblies are associated with reduced myotoxic activity. Finally, this study presents a toxin with novel functional/structural and evolutionary characteristics which can contribute for a more complete understanding of Lys49-PLA_2_s and development of structure-based drugs and other biotechnological products.

### Atomic coordinates

The MjTX-I coordinates and structure factors have been deposited in the Protein Data Bank with identification code 3T0R

## Supporting Information

Table S1 **Interfacial residues of the MjTX-I crystal structure.**
(DOC)

Table S2 **Interfacial salt bridges and hydrogen bonds of the MjTX-I crystal structure.**
(DOC)
