# Supplementary material for: Structural and Phylogenetic Studies with MjTX-I Reveal a Multi-Oligomeric Toxin – a Novel Feature in Lys49-PLA2s Protein Class
Source: PLoS One. 2013 Apr 3;8(4):e60610. doi: 10.1371/journal.pone.0060610 (PMC3616104; doi:10.1371/journal.pone.0060610)
Supplement: Table S1 — Interfacial residues of the MjTX-I crystal structure. (DOC) [file pone.0060610.s001.doc]

| Interface A/B | Interface A/C | Interface A/D | Interface B/D | Interface C/D |
| --- | --- | --- | --- | --- |
| L10 (B) | N60 (A)* | D76 (A/D)* | L58 (D) | K7 (C) |
| Q11 (A/B) | N88 (A) | K78 (A/D) | T59 (D) | L10 (C) |
| E12 (A/B) | P90 (A) | V83 (D) | N60 (D)* | Q11 (C/D) |
| T13 (A/B) | K110 (C)* | E86 (A) | P90 (D) | E12 (C/D) |
| G15 (A/B) | G111 (C) | E87 (D)* | C91 (D) | T13 (C/D) |
| W77 (A/B) | T112 (C) |  | K110 (B)* | G15 (C/D) |
| N79 (A/B) | Y113 (C) |  | G111 (B) | D76 (C)* |
| K80 (A/B) | N114 (C) |  | Y113 (B) | W77 (C/D) |
| T81 (A/B) | R117 (C)* |  | N114 (B) | K78 (D) |
| I82 (A) |  |  | K115 (B) | N79 (C/D) |
| I104 (A/B) |  |  | K116 (B) | K80 (C/D) |
| R107 (A/B) |  |  | Y120 (B) | T81 (C/D) |
| E108 (A/B) |  |  |  | I104 (D) |
| K110 (A/B)* |  |  |  | R107 (C/D) |
|  |  |  |  | K110 (C)* |

* Exclusive residues of MjTX-I (see Figure 6).
