# Supplementary material for: Structural and Phylogenetic Studies with MjTX-I Reveal a Multi-Oligomeric Toxin – a Novel Feature in Lys49-PLA2s Protein Class
Source: PLoS One. 2013 Apr 3;8(4):e60610. doi: 10.1371/journal.pone.0060610 (PMC3616104; doi:10.1371/journal.pone.0060610)
Supplement: Table S2 — Interfacial salt bridges and hydrogen bonds of the MjTX-I crystal structure. (DOC) [file pone.0060610.s002.doc]

| Interaction | Residues/involved atoms | Distance (Å) |
| --- | --- | --- |
| Hydrogen bonds | A: Q11 [ O ]; B: W77 [ NE1 ] | 2.77 |
|  | A: E12 [ OE2 ]; B: K80 [ NZ ] | 2.71 |
|  | A: E12 [ O ]; B: W77 [ NE1 ] | 3.56 |
|  | A: K80 [ O ]; B: K80 [ NZ ] | 2.76 |
|  | A: W77 [ NE1]; B: Q11[ O ] | 3.13 |
|  | A: K80 [ NZ ]; B: E12 [ OE2 ] | 2.60 |
|  | A: W77 [ NE1]; B: E12[ O ] | 3.48 |
|  | A: K80 [ NZ ]; B: K80 [ O ] | 2.66 |
|  | C: W77 [ NE1 ]; D: Q11[ O ] | 2.95 |
| Salt bridges | A: E12 [ OE1 ]; B: K80 [ NZ ] | 3.47 |
|  | A: E12 [ OE2 ]; B: K80 [ NZ ] | 2.71 |
|  | A: K80 [ NZ ]; B: E12 [ OE1] | 3.21 |
|  | A: K80 [ NZ ]; B: E12 [ OE2 ] | 2.60 |
|  | C: E12 [ OE1 ]; D: K80 [ NZ ] | 2.88 |
|  | C: E12 [ OE2 ]; D: K80 [ NZ ] | 2.39 |
|  | C: K80 [ NZ ]; D: E12 [ OE2 ] | 3.70 |
